# Spatiotemporal dataset on Chinese population distribution and its driving factors from 1949 to 2013

**DOI:** 10.1038/sdata.2016.47

**Published:** 2016-07-05

**Authors:** Lizhe Wang, Lajiao Chen

**Affiliations:** 1 School of Computer Science, China University of Geosciences, Wuhan, Hubei 430074, P.R. China; 2 Institute of Remote Sensing and Digital Earth, Chinese Academy of Sciences, Haidian, Beijing 100094, P.R. China

**Keywords:** Socioeconomic scenarios, Sustainability, Geography

## Abstract

Spatio-temporal data on human population and its driving factors is critical to understanding and responding to population problems. Unfortunately, such spatio-temporal data on a large scale and over the long term are often difficult to obtain. Here, we present a dataset on Chinese population distribution and its driving factors over a remarkably long period, from 1949 to 2013. Driving factors of population distribution were selected according to the push-pull migration laws, which were summarized into four categories: natural environment, natural resources, economic factors and social factors. Natural environment and natural resources indicators were calculated using Geographic Information System (GIS) and Remote Sensing (RS) techniques, whereas economic and social factors from 1949 to 2013 were collected from the *China Statistical Yearbook* and *China Compendium of Statistics from 1949 to 2008*. All of the data were quality controlled and unified into an identical dataset with the same spatial scope and time period. The dataset is expected to be useful for understanding how population responds to and impacts environmental change.

## Background & Summary

Human population distribution is described as the study of where people live. It is a key issue of population geography, focusing on the quantity of people, their spatial distributions and the resulting density over an area^1–4^. The research of population distribution is important, as it is concerned with how humans are related to and interact with their physical and socioeconomic environments^5,6^. Understanding what determines population distribution is fundamental to understanding the relationships between humans and the environment^7^. Population distribution studies encompass several different topics, including a population’s characteristics, patterns and rules, as well as how these changes over time and space^8,9^. Among these topics, what causes population distributions to be as they are—in other words, the relationship between population distribution and environmental factors—is the most important issue. Much attention has been paid to the relationship between population and its driving factors, including resources, environment and socio-economic development^10,11^. Several projects have set out to study the population-environment relationship, such as PERN (Population-Environment Network), launched in 2001 by the International Union for the Scientific Study of Population (IUSSP) (https://www.populationenvironmentresearch.org/home). Due to the complex interactions among population, resources, environment and socio-economic development, it is important to examine mechanisms of population distribution and evolution in order to deepen our understanding of spatial demography and support population policy- making.

The 20th century witnessed global population growth at an unprecedented rate. This has created enormous pressure on natural resources and the environment worldwide, such as agricultural production, fresh water stores and energy production^12,13^. Human overpopulation, which refers to the number of people exceeding the carrying capacity of a region, is one of the most serious problems humans and the environment face. On June 24, 2015, the world’s human population was about 7.253 billion as estimated by the United States Census Bureau. In addition, world population distribution is highly uneven, with some small areas featuring high population densities, while others are sparsely populated. For instance, the United Nations has expressed concern about continued excessive population growth in sub-Saharan Africa^14^. Therefore, solving human population problems and achieving a harmonious balance between population growth and the environment have become the most urgent issue faced by many countries.

Given all this, knowledge of the relationship between human populations and environmental factors, social conditions, and economic conditions is crucial in order to better understand and respond to the population problems associated with social, political and environmental problems. Data-driven spatio-temporal analysis and modeling of population distribution are essential ways of acquiring such knowledge. It is well established that much of our understanding of the relationship among spatial phenomena is inexplicitly embedded in spatio-temporal data. Therefore, both data-driven spatio-temporal analysis and modeling require detailed spatial information on populations, resources and environmental and socio-economic factors on a large scale and over the long term. Such datasets can be used to test and verify population modeling, develop new indicators of population distribution and examine the correlation between population distribution and driving factors, etc.^15–18^. Unfortunately, spatio-temporal data on populations and their driving factors on a large scale and over the long term is often difficult to obtain directly.

There are a handful of open sources of population related databases in China. Most of those databases mainly focus on social or economic aspects of population. Certain information about the natural environment that might be important influencing factors of population dynamics is absent in those databases. For instance, China Data Center, maintained by the University of Michigan, provides overall historical and social science data for China (http://chinadatacenter.org/default.aspx). From this data center, we can find very detailed population census data and Chinese statistical data. However, China Data Center does not provide any key natural environmental data, such as information about climate, topography and vegetation. This is also true of other similar databases. For example, other databases might also provide population-related datasets but with incomplete data items related to population dynamics and socio-economic data. In addition, those datasets usually only offer a short time period of data, often covering only one year or five years. Data Sharing Infrastructure of Earth System Science (http://www.geodata.cn), offered by the Institute of Geographic Sciences and Natural Resources Research, Chinese Academy of Sciences, also provides population-related data, such as population distribution data and natural resources data. However, it is a huge database focused on the interaction of earth systems; in other words, population and its dynamic is only a small part of the database. Therefore, the population-related database is incomplete and nonsystematic. Another example is the Thematic Database for Human-Earth System (http://www.data.ac.cn/index.asp), which is also housed at the Institute of Geographic Sciences and Natural Resources Research, Chinese Academy of Sciences. It supplies comprehensive datasets with respect to human and earth systems, including data related to global change, the natural environment, resources, disasters, population, economy, society, etc. However, most of the statistical data items only cover a short-term period. For example, the social and economic indicators only cover a time period from 2003 to 2008. In summary, the existing population-related databases are incomplete, with either incomplete data of environmental conditions or incomplete data of population and socio-economic factors.

In view of this, this study presents a long-term comprehensive spatio-temporal dataset of China’s population. The dataset is systematically generated based on the theory of push-pull migration law, which considers migration as an individual’s response to regional differential conditions. Push-pull indicators of population selected in this dataset are based on population redistribution theory. Data items, especially the natural environmental condition data, are generated according to frontier theories in their respective fields. The uniqueness of this dataset is that it is a thorough and compressive population database that focuses on the spatio-temporal dynamic of population and its potential spatio-temporal driving factors including detailed natural and socio-economic factors within China. Compared with the existing population-related databases for China mentioned above, this dataset provides overall population-related data, which not only includes detailed environmental condition data, but also consists of complete population and socio-economic data over a long-term time period. Such a dataset will be useful to understand how populations respond to environmental change and the influence of the environment and resources.

The main limitation of this study is that most population and socio-economic statistical data are at the province level. Even though they can capture the interprovincial differences of populations, such kinds of data are too coarse for detailed spatial analysis. County-level data about population are provided to present the spatial distribution of population; however, due to the limited data availability, we only obtained county-level data for a short period. In addition, due to the change of statistical indicators such as household registration population and usual resident population, the time periods of the data items remain inconsistent.

## Methods

### Theoretical basis of population distribution and dynamic analysis

Population growth primarily refers to natural and mechanical growth. Natural growth represents the net of births and deaths in a country’s population and does not take into account migration. In contrast, mechanical growth takes migration and its many potential motives into account. Migration, the movement or relocation of people, can be internal or external, voluntary or forced. Efforts have been made to methodologically quantify, measure and predict factors that play roles in human migration. Many laws of migration have been proposed, and Lee’s pull-push model developed in 1966 is one of the most important migration laws^19,20^. Lee classified factors as either positive or negative, and the migration process is governed by these ‘pull’ and ‘push’ factors. These factors can be economically, politically, culturally, or environmentally based. Push factors are negative conditions that can drive a person to move away from his original location and are often forceful incentives to migrate. Push factors include unemployment, lack of opportunities, natural disasters, severe environmental conditions, poor medical care, low security, and so on. Pull factors, on the other hand, are attractions that draw a person to move to a particular location. Better wages, more jobs, better education, and better medical care, are all examples of pull factors. To migrate, people can either be pushed from an unattractive location or find another place so attractive that they feel pulled toward it.

### Population distribution and dynamic data collection

This dataset contains 65-years’ time serial data for the whole of China (unit: million persons) and for each province (unit: 10,000 persons). In addition, spatial distribution of population at 1995, 2000, 2005 and 2010 with a resolution of 1 km is also provided (Data Citation 1). Due to the *hukou* (*household registration*) system in China, there are two different indicators to represent regional population. One is ‘household registration’ population (*huji renkou*), which only enumerates people who have a local *hukou* in the survey location (such as a city or a county). The other one is ‘usual resident’ population (*changzhu renkou*), counting the population of those without a local *hukou* but who have lived in a survey location for more than six months. Data in 1981 and before were taken from the statistics of household registration, while data after 1981 were taken from the statistics of usual residents. Due to the two different statistical indicators, there is a certain gap between the data before and after 1981. Such an inconsistency is a critical discrepancy of population data. However, before 1981 population migration among provinces in China was strictly controlled by the *hukou* system, which may result in low floating population (*liudong renkou*). Hence, the household registration population is close to the usual resident population, reducing the inconsistency of the data.

Data in 1953, 1964, 1982, 1990, 2000, and 2010 were drawn from the national population census, while the rest of the data were estimated from the nationwide annual population surveys. Data in 1987, 1995 and 2005 were estimated based on the National 1% Population Sample Survey, and were estimated based on the National Sample Survey on Population in other years. Data in 1982–1989 and 1990–1999 were adjusted based on the National Population Census in 1990 and 2000, respectively. Data for Beijing in 2006–2009 and data for Tibet in 2001–2009 have been adjusted according to the 2010 National Population Census in 2012. National population censuses before 2000, including 1953, 1964, 1982, and 1990 were conducted on July 1, while censuses in 2000 and 2010 were conducted on November 1. The rest of the data, estimated from the nationwide annual population surveys, were collected at year-end. The national total population did not include the population of Hong Kong Special Administrative Region, Macao Special Administrative Region and Taiwan Province. The military personnel of the Chinese People’s Liberation Army were included in the national total population.

The source data were originally collected from the *China Statistical Yearbook* from 1991 to 2013 and from the *China Compendium of Statistics from 1949 to 2008*. Data from the *China Statistical Yearbook of 1981 to 1995* were obtained from the printed book at the National Library and typed into Microsoft Excel, while data covering the years 1996 to 2014 is available from the website of the National Bureau of Statistics (http://www.stats.gov.cn/tjsj/ndsj/). The interpolation data from 1995, 2000, 2005 and 2010 was obtained from the Data Center for Resources and Environmental Sciences, Chinese Academy of Sciences (RESDC) (http://www.resdc.cn).

### Push-pull factor selection

In setting up our analysis, we selected the push-pull factors that are most likely to motivate migration. The potential push-pull factors used in the existing study include those relating to natural environment, natural resources, economy and society. To better characterize these factors on spatial and temporal scales, we selected quantified indicators first. The push-pull factors and these indicators are listed in Table 1.

### Process and calculation of push-pull indicators

#### Natural environmental factors

Natural environmental factors include climate factors, topographical factors, vegetation factors, and natural resources factors (Data Citation 1).

**Figure 1:**
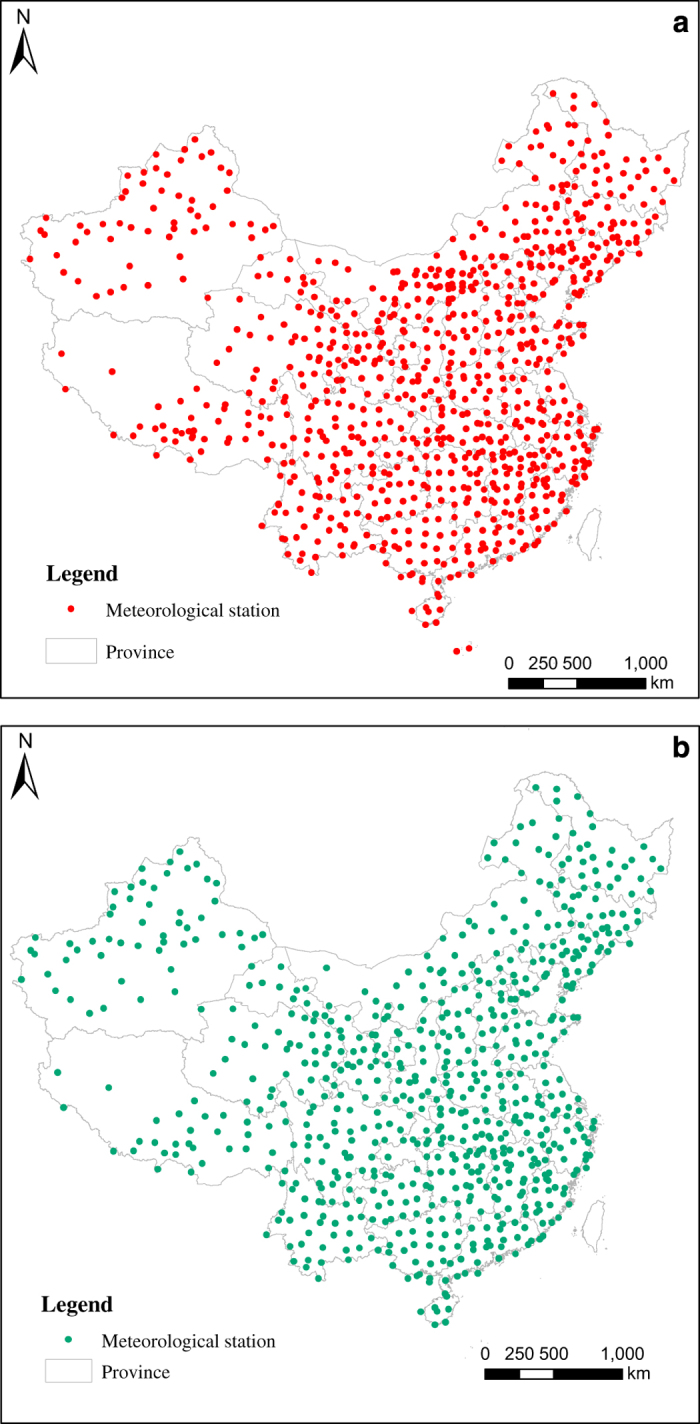
Distribution of meteorological stations. (**a**) Distribution of all meteorological stations (Taiwan is excluded). (**b**) Distribution of quality-controlled meteorological stations (Taiwan is excluded).

Climate factors. Climate factors include average annual temperature, relative humidity and precipitation, as well as a climate suitability index. As there is no regional climate data covering the period of our study, we had to interpolate the measurements from 846 meteorological stations in China^21^. The distribution of the original meteorological stations is shown in Fig. 1a. In accordance with standard data quality controls such as data cleaning, 161 meteorological stations were removed, and 685 of the stations (Fig. 1b) remained for data interpolation.

Average annual temperature, relative humidity and precipitation were first calculated for the time period of 1960 to 2011. The station-based average data were then subjected to existing data interpolation techniques, including Inverse Distance Weighted (IDW), Spline, Ordinary Kriging and CoKriging. Since no information is available about which method could be the optimal one for climate factors interpolation in China, cross-validation is used to estimate accuracy and evaluate the performance of each method. Cross validation is a model validation technique which partitions a sample of data into *known data* (training dataset) and *unknown data* (testing dataset), performing analysis with *known data* and validating the analysis with the unknown data^22^. In this way, validation is independent and thus can overcome the problem of overfitting. In this study, we remove one data point at a time and use the rest of the data for interpolation^23^. The climate variables in the location of the removed point are estimated from the remaining samples and then compared with the actual value of this point. This process is repeated until every sample point has been removed. The performance of the interpolation is then calculated using the Root-Mean-Squared Residuals (RMSE) as follows:

$$\text{RMSE}=\sqrt{\frac{\sum_{i=1}^{n}\left(C_{i\_si\;m}-C_{i\_obs}\right)}{n}}$$

Where, $C_{i\_si\;m}$ is the simulated value of climate variable at the point of *i*, $C_{i\_obs}$ is the value of observed value of climate variable at the point of *i*, and *n* is the number of the data point, the number of the meteorological station.

After our measurement data were interpolated, we calculated the indicator of climate suitability index. The Temperature Humidity Index (THI) was used to represent a climate suitability index based on Feng’s study^24^. The concept of THI was originally proposed by Thom^25^ to quantify climate suitability. Such an index can better represent the impact of human settlement on the climate compared with other standards of measurement, such as the wind speed index or the human comfort index. The index was calculated according to the following equation:

TH=1.8T−0.55×(1−RH)×(1.8T−26)

Here, *T* is the average temperature and H is the relative humidity.

Both interpolation of the climate data and the calculation of THI in this study were done using the Spatial Analyst module of ArcGIS 10.1.

Topographical factors. To quantitatively represent topographical conditions, Digital Elevation Model (DEM) and Relief Degree of Land Surface (RDLS) were selected. The DEM data was provided by the Cold and Arid Regions Sciences Data Center at Lanzhou (http://westdc.westgis.ac.cn).

To synthetically represent the altitude and incision depth of a region, RDLS is an effective index for describing the landform at a macro-scale, and it has been widely used for this purpose. It is calculated by the following equation, which was proposed by Feng^26^:

RDLS=ALT/1000+((Max(H)−Min(H))×(1−P(A)/A))/500

Here, *ALT* is the average elevation of the neighborhood, and *Max(H)* and *Min(H)* are the maximum and minimum heights of the neighborhood, respectively. Further, *P(A)* is the area of ground examined, and A is the area of the neighborhood. In this study, *A* is set to 25 km^2^. A 10 km×10 km window was established as the extraction neighborhood unit, and this study extracted the *Max(H)* and the *Min(H)* within that window. Each window produced two data layers, and the *Min(H)* was subtracted from the *Max(H)* to obtain the height difference of each grid.

Vegetation factors. The Normalized Differential Vegetation Index (NDVI) is used to represent the land use and land cover condition. NDVI is defined as the difference between near-infrared and red reflectance divided by the sum of the two as follow^27^:

$$NDM=\frac{\left(N\;R-RED\right)}{\left(N\;R+RED\right)}$$

where NIR is the spectral reflectance measurement of near-infrared red light while RED is the spectral reflectance measurement of red light.

NDVI is closely linked to vegetation cover, due to the fact that chlorophyll absorbs red light while the mesophyll leaf structure scatters near-infrared red light^27,28^. A higher value of NDVI represents more extensive coverage by vegetation. A negative value of NDVI represents the absence of vegetation.

NDVI can be derived from remote sensing data, and there are existing NDVI products we can directly obtain from the data center. In this study, the NDVI data include the Global Inventory Modelling and Mapping Studies (GIMMS) NDVI data from 1981 to 2006 and the Moderate-Resolution Imaging Spectroradiometer (MODIS) NDVI data from 2007 to 2010.

GIMMS NDVI datasets were generated to provide records of monthly changes in terrestrial vegetation with a spatial resolution of 8 km (ref. 29). These datasets can provide long-term time serial NDVI data since 1981. MODIS NDVI datasets were generated using a monthly time scale with a spatial resolution of 250 m (ref. 30). To eliminate spatial mismatch of the two datasets, we have downscaled the spatial resolution of MODIS NDVI to 1 km and upscaled the spatial resolution of GIMMS NDVI to 1 km. As both datasets were obtained monthly, we aggregated the monthly data into yearly data.

GIMMS-derived NDVI data was obtained from the Environmental and Ecological Science Data Center for West China (Cold and Arid Regions Sciences Data Center). MODIS-derived NDVI were obtained from the LAADS Web of Goddard Space Flight Center, National Aeronautics and Space Administration (NASA).

#### Natural resources factors

Natural resources considered in this study include water, forest and land resources (Data Citation 1). We chose the amount of water resources and Net Primary Productivity (NPP) as indicators to characterize natural resources. The spatial distribution of the amount of water resources could not be directly acquired, so we obtained data for the water resources of every city in China from the *China Statistical Yearbook*, which had to be redeveloped into spatial distribution data. The NPP dataset was acquired from the LAADS Web of Goddard Space Flight Center, NASA. Data on the quality of cultivated land in China was obtained from the Data Sharing Infrastructure of Earth System Science.

#### Economic pull-push factors

The economic factors considered in the present study include data items relating to gross domestic product (GDP), primary industry, secondary industry, tertiary industry and total investment in fixed assets (Data Citation 1). The province-based data of these five indicators were collected from the *China Statistical Yearbook* from 1981 to 2014 and *China Compendium of Statistics from 1949 to 2008*.

#### Social pull-push factors

Social pull-push factors mainly fall into six categories: food, traffic, education, technology, health and medical conditions and human living conditions (Data Citation 1). These factors are based on indicators of total grain production, length of railways, length of highways, length of navigable inland waterways, number of regular primary schools, number of higher education institutions, number of patent applications, number of health agencies, number of beds in health care agencies, per capita annual income of urban households, per capita annual income of rural households, Engel’s coefficient of urban households and Engel’s coefficient of rural households. The province-based data of these twenty indicators were collected from the *China Statistical Yearbook* for the years from 1981 to 2014 and *China Compendium of Statistics from 1949 to 2008*.

## Data Records

### Data record 1

The spatial dataset provided in grid format includes the data file on population, environment and natural resources. The data are expressed in raster format, with a resolution of 1 km.

### Data record 2

The dataset containing the social and economic factors at province scale is provided in XLS files and in shapefile format.

## Technical Validation

The quality of all data was controlled to ensure that the entries represent true trait variation. The data entries that we collected from data centres, such as population distribution with 1 km, were checked by the data collectors. We validated the data we produced by cross validation and by examining the spatial distribution based on existing theory on democracy, environment, society and economy. We used the cross validation approach to assess the accuracy of the climate data entries that we interpolated from meteorological stations. For precipitation interpolation, the mean of the prediction error is about 0.88, the root-mean-square of the prediction error is about 151.94, and the root-mean-square of the standardized prediction error is about 0.82. For temperature interpolation, the mean of the prediction error is about −0.04, the Root-Mean-Square of the prediction error is about 2.19, and the Root-Mean-Square of the standardized prediction error is about 1.10. For relative humidity interpolation, the mean of the prediction error is about 0.05, the root-mean-square of the prediction error is about 4.21, and the root-mean-square of the standardized prediction error is about 3.86. The prediction errors of temperature, precipitation, and relative humidity are acceptable. For the other data items we produced (e.g., Temperature Humidity Index (THI), Topographic, Relief Degree of Land Surface (RDLS)), we validated the spatial distribution according the existing knowledge about the Chinese environment, society and economy. For the economic and social data we collected from the *China Statistical Yearbook* for the years 1981 to 2014 and *China Compendium of Statistics from 1949 to 2008*, we have checked all of the records, and those that are inconsistent are removed. The on-line database may also be corrected if new information becomes available.

## Usage Notes

To demonstrate how to use the dataset we produced, we conducted a case study to analyse the relationship between population and driving factors in China. The population distribution of China is dramatically uneven. Hu Huanyong divided the area of China into two roughly equal parts by drawing the Heihe-Tengchong line in 1935 (refs 31–33). On the east side of the line, 96% of the population lives in the region that accounts for 42.9% of the land area of China; on the west side of the line, only 4% of the population lives in the region that accounts for 57% of the land area of China. Although more than 80 years have passed since 1935, the modern statistics remain very close to the original number of population. Then the question arises: What is the intrinsic mechanism that dictates population distribution, and can we quantify the mechanism so as to better understand how the Heihe-Tengchong Line responds to environmental and socio-economic change? To answer these questions, we conducted a case study to analyse the driving factors affecting the Heihe-Tengchong Line and to assist in population policymaking.

### Spatio-temporal dynamics of population distribution over 80 years in China (1933 to 2013)

**Figure 2:**
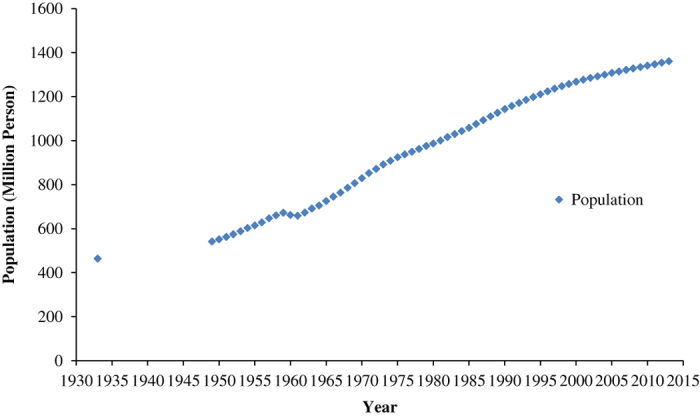
Demographic Evolution of China in 1933–2013 (Taiwan is excluded). Blue point represents the amount of population of each year.

**Figure 3:**
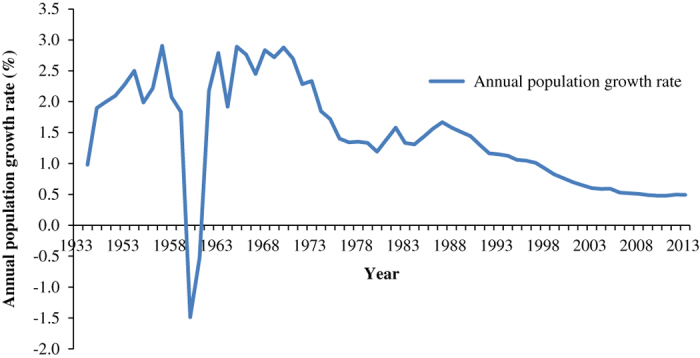
Annual Population Growth Rate of China in 1933–2013 (Taiwan is excluded). Blue line is the annual population growth rate of China from 1933 to 2013.

**Figure 4:**
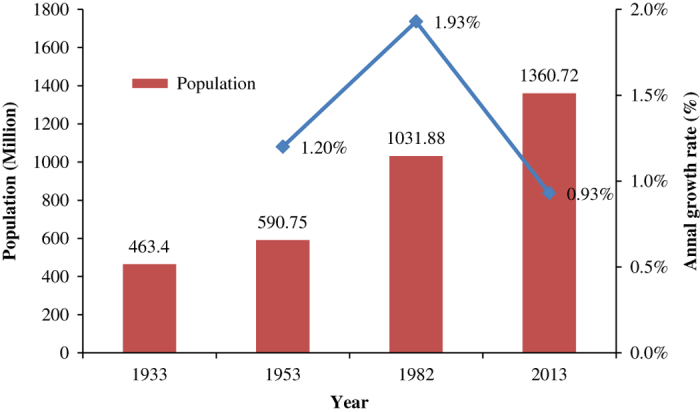
Demographic Evolution of China in 1933–2013 (Taiwan is excluded). Blue line is the annual growth rate of population, red column is the amount of population of year 1933, 1953, 1982 and 2013.

Demographic evolution and average annual population growth from 1933 to 2013 are shown in Figs 2, 3 and Table 2. The total population in 1933 was 463 million, with a population density of 48 persons per square kilometer^31^. During the eight decades from 1933 to 2013, the average annual growth rate was approximately 1.36%. In 2013, the total population of China was 1361 million, with a net increase of 32% and an average annual growth rate of 1.36% since 1933. We divided the larger period of 1933 to 2013 into three sub-periods: 1933 to 1953, 1953 to 1982, and 1982 to 2013. The rate of population growth was much faster during the period 1953 to 1982 than those rates in the periods 1933 to 1953 and 1982 to 2013, with a net increase of 75% and an average annual growth rate of 1.93% (Fig. 4). This is due to the political stability and rapid development of the economy after the liberation of China. However, there is a sharp decrease between 1958 and 1961, which can be attributed to a three-year period of economic difficulties in China. In the period 1933 to 1953, the population grew more slowly than it did from 1953 to 1982 because of the Anti-Japanese and National Liberation Wars. In the period from 1982 to 2013, the population grew slowly due to the ‘one-child policy’, which caused a drastic decline in the birth rate and the natural rate of growth.

**Figure 5:**
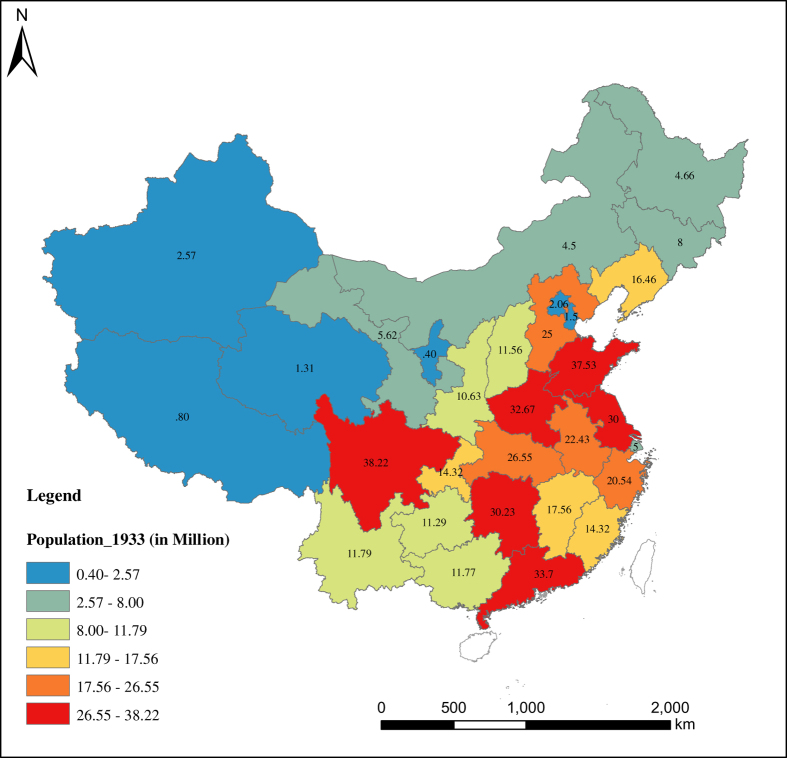
Population Distribution of China in 1933 (Taiwan and Hainan are excluded). Different value of population is expressed by different color group and population of each province is labeled on the map.

**Figure 6:**
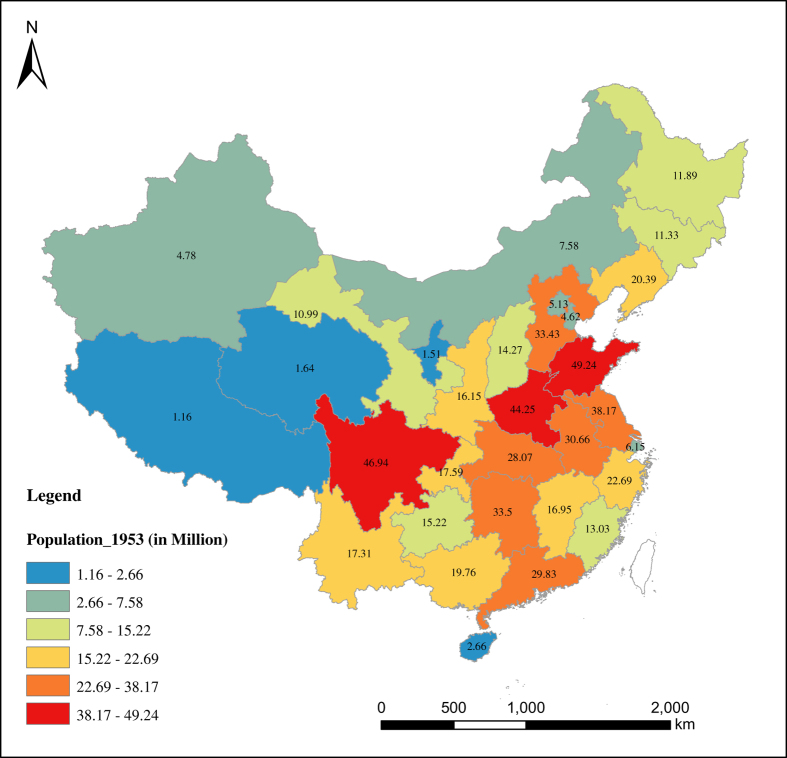
Population Distribution of China in 1953 (Taiwan is excluded). Different value of population is expressed by different color group and population of each province is labeled on the map.

**Figure 7:**
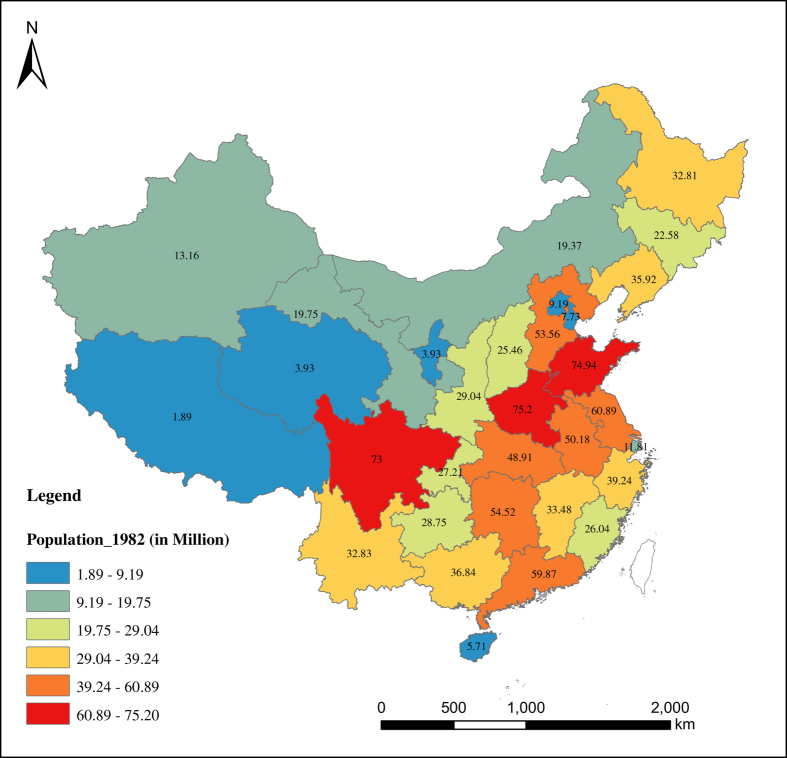
Population Distribution of China in 1982 (Taiwan is excluded). Different value of population is expressed by different color group and population of each province is labeled on the map.

**Figure 8:**
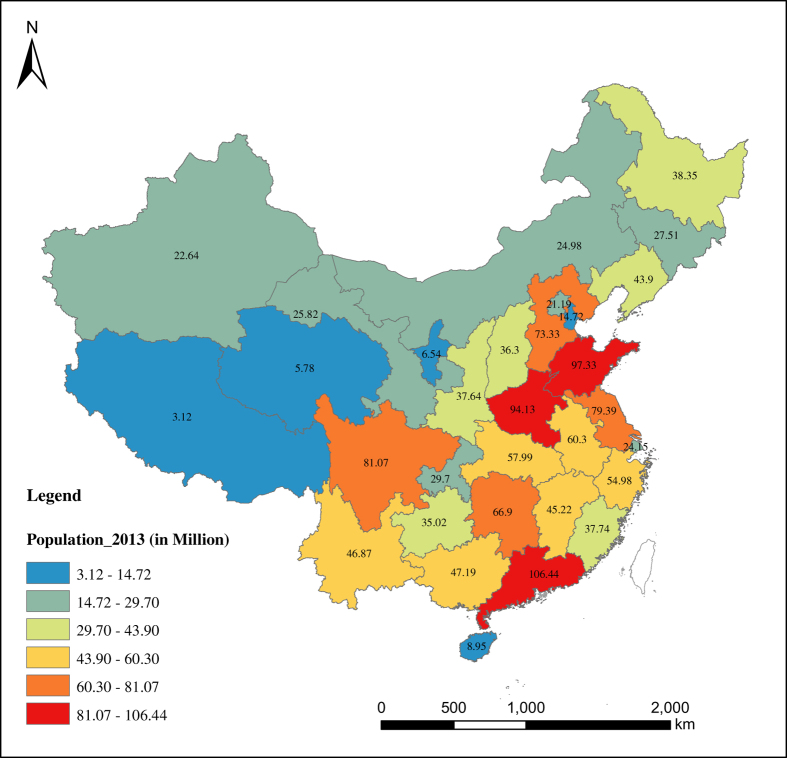
Population Distribution of China in 2013 (Taiwan is excluded). Different value of population is expressed by different color group and population of each province is labeled on the map.

With respect to the spatial distribution of China’s population, the pattern reflects a great difference between the southeast and northwest parts of the country (Figs 5,6,7,8). Most of the population is concentrated in the southeastern side of China. Guangdong has been the most populous province with a population of 106.44 million in 2013, while Tibet is the least populous province with a population of 3.12 in 2013. The spatial distribution of the population has not changed much over the eight decades from 1933 to 2013. From Figs 5,6,7,8, we see that the rate of population growth varied in different provinces. Assuming that the average annual growth rate of China as a whole represents the natural growth of the country, the province with an annual population growth rate higher than that of the whole of China is the region with the most immigration. From Supplementary Figs S1–S3, we can see that Beijing and Tianjin received the most immigrants over the eight decades from 1933 to 2013. During the period 1933 to 1953, Ningxia (6.87%), Tianjin (5.79%), Beijing (4.67%), Heilongjiang (4.79%), and Gansu (3.41%) were the regions that experienced the most inbound immigration, while southeastern provinces such as Guangdong (−0.61%), Fujian (−0.47%), Jiangxi (−0.18%) and Hubei (0.28%) were the main migrant regions. During the period 1953 to 1982, Heilongjiang (3.56%), Xinjiang (3.55%), Ningxia (3.35%) and Inner Mongolia (3.06%) experienced more inbound immigration than other regions. In this period, especially from the late 1950s to the early 1980s, population migration among provinces was strictly controlled by the *hukou* system; therefore, the government’s central political policy is the main driving factor of population movement. One example is the policy of ‘Countryside Movement’ during 1960 to 1970, which encouraged educated youth to countryside and remote areas. Such movement has seriously changed the distribution of human resources among the provinces, which led to the population increasing in western provinces and decreasing in coastal provinces^34^. For the last three decades, from 1982 to 2013, the immigrant-migrant pattern changed considerably. In this period, population migration was mainly controlled by regional economic development. The most immigrated-to provinces were Beijing (2.73%), Shanghai (2.33%), Tianjin (2.1%), and Guangdong (1.87%), while the top migrant provinces were Chongqing (0.28%), Sichuan (0.34%) and Heilongjiang (0.50%), Hubei (0.55%) and Anhui (0.59%), which experienced lower growth than the average annual population growth rate of 1.0%. Tibet has a higher population growth rate in this period, which might be due to the ‘one child’ policy rather than immigration. This is because the ‘one child’ policy only applies to the Han Chinese, so provinces with larger minority populations experienced higher population growth.

### Analysis of the relationship between population and driving factors

#### Driving factors of population evolution from 1949 to 2013

An analysis of population and driving factors in China as a whole from 1949 to 2013 was first conducted using the correlation approach. Potential driving factors were determined using the following 20 indicators: GDP X1 (100 million yuan), primary industry X2 (100 million yuan), secondary industry X3 (100 million yuan), tertiary industry X4 (100 million yuan), total investment in fixed assets X5 (100 million yuan), total grain products X6 (millions of tons), number of health care agencies X7 (units), number of beds in health care agencies X8 (910,000 beds), length of transport routes X9 (10,000 km), length of railways X10 (10,000 km), length of highways X11 (10,000 km), length of navigable inland waterways X12 (10,000 km), number of schools of higher education X13 (units), number of regular primary schools X14 (units), number of three kinds of patent applications accepted X15 (units), number of three kinds of patent applications granted X16 (units), per capita annual income of urban households X17 (yuan), per capita annual income of rural households X18 (yuan), Engel’s coefficient of urban households X19 and Engel’s coefficient of rural households X20.

A correlation matrix of potential driving factors at the province level in China is shown in Table 3. It indicates that X6: total grain products (0.98), X10: length of railways (0.96), X19: Engel’s coefficient of urban households (−0.95) and X20: Engel’s coefficient of rural households (−0.90) are the highest covariance indicators relating to population growth from 1949 to 2013 in China.

To eliminate the interaction among these factors and evaluate the individual contribution of each, a stepwise regression analysis was conducted. First, a multiple linear regression equation with seven driving factors was established. Table 4 shows the analysis of variance (ANOVA). The mean squared error term is smaller than that of the model, and the squared multiple correlation R^2^=147839.828/147994.825=0.999, indicating that 99.9% of the variability in the population is explained by the driving factors.

The driving factors selected by stepwise regression and their contribution are listed in Table 5. The standardized coefficient can represent the contribution of the factors. We can assert that the contribution of socio-economic factors is ranked as follows: number of regular primary schools X14 (−0.757), tertiary industry X4 (−0.637), number of beds in health care agencies X8 (0.427), per capita annual income of urban households X17 (0.407), length of railways X10 (0.36) and number of higher education institutions X13 (-0.015).

#### Driving factors of population spatial distribution

Natural driving factors. Spatial correlation analysis was conducted between the spatial distribution of population and natural geographic data, climate data, and natural resources. The data relating to population distribution in the year 2010 and the natural environment data presented in the above of this paper were used. For these nine indicators, the top five relevant indicators are listed in Table 6. The first important push-pull factor revealed was water resources, which has a correlation coefficient of 0.51 with population distribution. Climate factors, such as precipitation, temperature and relative humidity, were less closely correlated with population. Among these factors, average climate demonstrated a much higher correlation with population, which was 0.19.

When we analyzed the correlation of population and driving factors separately, we found that the driving factors were quite different among the provinces (Table 7). In water-limited regions, water is highly influential on population distribution. For example, in Beijing, the correlation of water resources and population was about 0.72, which is higher than the overall correlation coefficient of 0.46. This means restriction of water resources has a much higher impact on the population in Beijing than it does on other provinces.

Socio-economic driving factors. The correlation of socio-economic factors with population distribution in different provinces is shown in Table 8 (available *online only*). It illustrates that food was still the most important driving factor in undeveloped provinces. For example, in Gansu, Ningxia, Anhui, Guangxi, Guizhou, Yunnan, Hebei and Henan, the most important driving factor was total grain production. The second-most important factor in most of these provinces related to traffic. However, in developed provinces, such as Beijing, Shanghai, Jiangsu, Zhejing and Fujian, the most important driving factors were economic. For instance, GDP was the most important factor influencing the evolution of population distribution in Shanghai, while in Beijing the most important factor was secondary industry.

In summary, in the case study we demonstrated that the spatio-temporal dataset is helpful in analysing the relationship between human population and natural-socio-economic driving factors. Based on these analyses, we can generate knowledge of human population distribution and its evolution, which will assist us in resolving population problems and achieving harmony among humans, the environment, natural resources, ecology and so on.

## Additional Information

**How to cite this article:** Wang, L. & Chen, L. Spatiotemporal dataset on Chinese population distribution and its driving factors from 1949 to 2013. *Sci. Data* 3:160047 doi:10.1038/sdata.2016.47 (2016).

## Supplementary Material

Supplementary Information

## Figures and Tables

**Table 1:** Potential push-pull factors of population in China.

**Factors**	**Indicator**
Natural environmental factors	
Climate	Average annual temperature
	Average annual relative humidity
	Average annual precipitation
	Climate suitability index
Topography	DEM
	Relief Degree of Land Surface (RDLS)
Vegetation	NDVI
Natural resources	
Water resources	Amount of water resources
Forest resources	Net Primary Product (NPP)
Economic factors	
Overall GDP	Goss Domestic Product (GDP)
GDP classification	Primary industry
	Secondary industry
	Tertiary industry
Investment	Total investment in fixed Assets
Social factors	
Food	Total grain products
Health and medical condition	Number of health care agencies
	Number of beds in health care agencies
Traffic	Length of total transport routes
	Length of railways
	Length of highways
	Length of navigable inland waterways
Education	Number of higher education institutions
	Number of regular primary schools
Technology	Number of three kinds of patent applications accepted
	Number of three kinds of patent applications granted
People’s Living Condition	Per capaita annual income of urban household
	Per capaita annual income of rural household
	Engel’s coefficient of urban household
	Engel’s coefficient of rural household

**Table 2:** Demographic Evolution of China’s Provinces in 80 years (1933–2013).

	**Population of 1933 (Million)**	**Population of 1953 (Million)**	**Population of 1982 (Million)**	**Population of 2013 (Million)**	**Average annual growth rate of 1933–1953 (%)**	**Average annual growth rate of 1953–1982 (%)**	**Average annual growth rate of 1982–2013 (%)**	**Average annual growth rate of 1933–2013 (%)**
Beijing	2.06	4.14	9.23	21.15	3.53	**2.8**	**2.80**	2.95
Tianjin	1.5	2.69	7.76	14.72	2.95	**3.73**	**2.16**	2.90
Hebei	25	36.14	53	73.33	1.84	1.31	1.09	1.35
Shandong	37.53	48.88	74.41	97.33	1.32	1.45	0.90	1.20
Henan	32.67	44.21	74.42	94.13	1.51	1.81	0.79	1.33
Liaoning	16.46	20.56	35.72	43.90	1.12	1.93	0.69	1.23
Jilin	8	11.29	22.56	27.51	1.73	2.42	0.66	1.56
Heilongjiang	4.66	11.9	32.66	38.35	**4.79**	3.55	0.54	2.67
Shanxi	11.56	14.31	25.29	36.30	1.08	1.99	1.21	1.44
Shannxi	10.63	15.88	28.9	37.64	2.01	2.09	0.88	1.59
Gansu	5.62	11.59	19.57	25.82	3.68	1.83	0.93	1.92
Ningxia	0.4	1.94	3.9	6.54	**8.21**	2.44	1.74	3.55
Shanghai	5	8.5	11.86	24.15	2.69	3.02	2.40	1.99
Jiangsu	30	38.4	60.5	79.39	1.24	1.57	0.91	1.22
Anhui	22.43	30.66	49.67	60.30	1.59	1.68	0.65	1.24
Jiangxi	17.56	16.77	33.19	45.22	-0.2	2.38	1.04	1.19
Hubei	26.55	27.79	47.8	57.99	0.24	1.89	0.65	0.98
Hunan	30.23	33.23	54.01	66.91	0.48	1.7	0.72	1.00
Zhejiang	20.54	22.87	38.89	54.98	0.54	1.85	1.16	1.24
Fujian	14.32	13.14	25.93	37.74	-0.43	2.37	1.26	1.22
Taiwan	5	7.59	18.27	0.00	2.11	3.08		
Guangdong	33.7	36.74	59.3	106.44	0.43	1.66	1.97	1.45
Guangxi	11.77	17.59	36.42	47.19	2.01	2.54	0.87	1.75
Sichuan	52.54	65.68	99.71	81.07	1.12	1.45	-0.69	0.54
Guizhou	11.29	15.04	28.55	35.02	1.44	2.24	0.68	1.43
Yunnan	11.79	17.47	32.55	46.87	1.98	2.16	1.22	1.74
Inner Mongolia	4.5	7.33	19.27	24.98	2.47	3.39	0.87	2.17
Xinjiang	2.57	4.87	13.08	22.64	3.23	3.47	1.85	2.76
Qinghai	1.31	1.68	3.9	5.78	1.24	2.94	1.32	1.87
Tibet	0.8	1.27	1.89	3.12	2.35	1.38	1.68	1.72
Whole China	463.4	590.75	1031.88	1360.72	1.2	1.93	0.93	1.36

**Table 3:** Correlation Matrix of Potential Driving Factors on Province-scale in China (1949–2013).

	**Y**	**X 1**	**X 2**	**X 3**	**X 4**	**X 5**	**X 6**	**X 7**	**X 8**	**X 9**	**X 10**	**X 11**	**X 12**	**X 13**	**X 14**	**X 15**	**X 16**	**X 17**	**X 18**	**X 19**	**X 20**
Y	1.00																				
X 1	0.66	1.00																			
X 2	0.74	0.99	1.00																		
X 3	0.67	1.00	0.99	1.00																	
X 4	0.64	1.00	0.98	1.00	1.00																
X 5	0.56	0.98	0.96	0.98	0.99	1.00															
X 6	0.98	0.71	0.79	0.72	0.69	0.63	1.00														
X 7	0.76	0.66	0.72	0.66	0.65	0.56	0.71	1.00													
X 8	0.95	0.83	0.88	0.83	0.82	0.77	0.96	0.77	1.00												
X 9	0.79	0.95	0.96	0.96	0.94	0.90	0.80	0.72	0.88	1.00											
X 10	0.96	0.85	0.89	0.85	0.83	0.77	0.96	0.81	0.99	0.91	1.00										
X 11	0.79	0.95	0.96	0.96	0.94	0.90	0.81	0.70	0.88	1.00	0.91	1.00									
X 12	-0.13	−0.04	−0.09	−0.04	−0.03	0.01	−0.19	0.29	−0.08	0.04	−0.04	0.02	1.00								
X 13	0.84	0.92	0.93	0.92	0.90	0.86	0.86	0.75	0.92	0.95	0.95	0.95	0.09	1.00							
X 14	−0.54	−0.76	−0.79	−0.77	−0.74	−0.69	−0.58	−0.33	−0.60	−0.71	−0.64	−0.71	0.07	−0.73	1.00						
X 15	0.72	0.98	0.96	0.97	0.99	1.00	0.84	0.55	0.99	0.90	0.94	0.90	0.73	0.91	−0.82	1.00					
X 16	0.71	0.98	0.96	0.97	0.98	0.99	0.84	0.59	0.99	0.89	0.94	0.89	0.72	0.90	−0.81	1.00	1.00				
X 17	0.82	1.00	1.00	1.00	0.99	0.97	0.85	0.77	0.96	0.96	0.98	0.96	0.41	0.96	−0.93	0.97	0.96	1.00			
X 18	0.83	0.99	1.00	0.99	0.99	0.97	0.87	0.81	0.97	0.95	0.98	0.94	0.40	0.94	−0.92	0.97	0.97	1.00	1.00		
X 19	−0.95	−0.80	−0.84	−0.80	−0.78	−0.70	−0.81	−0.89	−0.77	−0.84	−0.90	−0.83	−0.47	−0.85	0.96	−0.67	−0.66	−0.84	−0.84	1.00	
X 20	−0.90	−0.88	−0.90	−0.89	−0.87	−0.82	−0.83	−0.84	−0.86	−0.91	−0.95	−0.91	−0.44	−0.94	0.96	−0.83	−0.82	−0.91	−0.90	0.94	1.00

**Table 4:** Analysis of Variance (ANOVA).

	**Sum of square**	**DF**	**Mean square**	**F**	**Sig.**
Model	147839.828	7	21119.975	2043.917	0.000
Error	154.996	15	10.333		
Total	147994.825	22			

**Table 5:** Coefficient of the regression model.

	**Unstandardized Coefficients**		**Standardized Coefficients**	**t**	**Sig.**
	**B**	**Std.Error**			
**Number of regular primary school X14**	-3.727	0.387	−0.757	−9.642	0.000
**Number of higher education institutions X13**	−0.035	0.012	−0.150	−2.867	0.012
**Number of beds in health care agencies X8**	1.105	0.080	0.427	13.885	0.000
**Number of three kinds of patent applications accepted X15**	−8.584E-005	0.000	−0.215	−1.767	0.098
**Length of railways X10**	37.618	11.192	0.36	3.361	0.004
**Tertiary industry X4**	−0.002	0.000	−0.637	−3.516	0.003
**Per capaita annual income of urban household X17**	0.008	0.003	0.407	2.385	0.031

**Table 6:** Correlation coefficient of natural driving factors with population.

**Indicator**	**Amount of water resources**	**Average annual temperature**	**Relative Humidity**	**Climate suitability index**	**Precipitation**
**Pearson correlation coefficient**	0.46	0.16	0.16	0.15	0.15

**Table 7:** Correlation of natural environment factors with population in different provinces.

**Province**	**First relevant driving factor**	**Second relevant driving factor**	**Third relevant driving factor**
**Beijing**	Climate suitability index (0.89)	Amount of water resources (0.72)	Average annual relative humidity (0.66)
**Tianjin**	Average annual relative humidity (0.96)	Average annual precipitation (0.57)	Climate suitability index (0.56)
**Hebei**	Climate suitability index (0.56)	Amount of water resources (0.60)	Average annual temperature (0.51)
**Shandong**	DEM (0.91)	Climate suitability index (0.78)	Average annual precipitation (0.11)
**Henan**	Climate suitability index (0.95)	Amount of water resources (0.36)	-
**Liaoning**	Amount of water resources (0.51)	Average annual relative humidity (0.33)	Average annual precipitation (0.32)
**Jilin**	Amount of water resources (0.55)	RDLS (0.2)	Average annual precipitation (0.10)
**Heilongjiang**	Amount of water resources (0.3)	DEM (0.1)	RDLS (0.26)
**Shanxi**	Amount of water resources (0.72)	Climate suitability index (0.54)	Average annual temperature (0.37)
**Shaanxi**	Amount of water resources (0.50)	RDLS (-0.18)	Average annual precipitation (0.16)
**Gansu**	Average annual relative humidity (-0.66)	Climate suitability index (-0.63)	Amount of water resources (0.51)
**Ningxia**	Amount of water resources (0.43)	Climate suitability index (-0.38)	Average annual relative humidity (-0.26)
**Shanghai**	Amount of water resources (0.76)	Average annual relative humidity (0.74)	Climate suitability index (-0.57)
**Jiangsu**	Average annual temperature (0.83)	DEM (0.70)	Climate suitability index (0.63)
**Anhui**	Average annual relative humidity (0.49)	Amount of water resources (0.48)	Climate suitability index (0.42)
**Jiangxi**	Climate suitability index (0.99)	Average annual relative humidity (0.93)	Amount of water resources (0.72)
**Jiangxi**	Climate suitability index (0.99)	Average annual relative humidity (0.96)	-
**Hunan**	Amount of water resources (0.49)	Average annual relative humidity (0.15)	Average annual precipitation (0.14)
**Zhejiang**	Amount of water resources (0.42)	Average annual relative humidity (0.40)	Climate suitability index (0.35)
**Fujian**	Amount of water resources (0.59)	Average annual relative humidity (0.33)	Climate suitability index (0.31)
**Guangdong**	Amount of water resources (0.50)	Average annual relative humidity (0.41)	Climate suitability index (0.41)
**Guangxi**	Amount of water resources (0.52)	Average annual precipitation (0.27)	Average annual temperature (0.14)
**Sichuan**	Amount of water resources (0.73)	Climate suitability index (0.27)	Average annual temperature (0.14)
**Guizhou**	Amount of water resources (0.67)	Climate suitability index (0.19)	Average annual precipitation (0.19)
**Yunnan**	Amount of water resources (0.57)	NPP (0.16)	Average annual precipitation (0.14)
**Inner MongoliaInner Mongolia**	Climate suitability index (-0.35)	DEM (-0.35)	RDLS (-0.27)
**Xinjiang**	Average annual precipitation (-0.88)	DEM (-0.80)	Amount of water resources (0.58)
**Qinghai**	DEM (0.52)	Amount of water resources (0.42)	-
**Tibet**	RDLS (-0.68)	DEM (-0.50)	Amount of water resources (0.39)
**Whole China**	Amount of water resources (0.46)	Average annual relative humidity (0.16)	Average annual temperature (0.16)

**Table 8:** Correlation of social-economic factors with population in different provinces

**Province**	**First relevant driving factor**	**Second relevant driving factor**	**Third relevant driving factor**	**Forth relevant driving factor**	**Fifth relevant driving factor**
Beijing	Secondary industry (0.983)	Total investment in fixed Assets (0.982)	GDP (0.971)	Length of highways (0.964)	Tertiary industry (0.950)
Tianjin	Primary industry (0.970)	GDP (0.969)	Secondary industry (0.967)	Tertiary industry (0.962)	Length of railways (0.960)
Hebei	Total grain products (0.93)	Length of railways (0.91)	Number of beds in health care agencies (0.87)	Secondary industry (0.862)	GDP (0.827)
Shandong	Length of railways (0.88)	Primary industry (0.866)	Total grain products (0.852)	Number of higher education institutions (0.814)	Secondary industry (0.813)
Henan	Total grain products (0.795)	Length of railways (0.699)	Primary industry (0.683)	Number of higher education institutions (0.592)	Tertiary industry (0.581)
Liaoning	Primary industry (0.806)	Length of highways (0.773)	Tertiary industry (0.767)	GDP (0.759)	Number of beds in health care agencies (0.750)
Jilin	Length of navigable inland waterways (0.852)	Total grain products (0.839)	Primary industry (0.796)	Tertiary industry (0.738)	Length of highways (0.720)
Heilongjiang	Length of navigable inland waterways (0.89)	Total grain products (0.77)	Secondary industry (0.762)	GDP (0.709)	Tertiary industry (0.656)
Shanxi	Length of railways (0.900)	Length of highways (0.852)	Primary industry (0.848)	Number of beds in health care agencies (0.846)	Number of higher education institutions (0.832)
Shannxi	Length of navigable inland waterways (0.967)	Number of beds in health care agencies (0.783)	Primary industry (0.691)	Tertiary industry (0.643)	GDP (0.637)
Gansu	Total grain products (0.810)	Length of navigable inland waterways (0.724)	Number of beds in health care agencies (0.688)	Primary industry (0.648)	Length of railways (0.625)
Ningxia	Total grain products (0.978)	Number of beds in health care agencies (0.900)	Length of railways (0.888)	Primary industry (0.859)	Number of higher education institutions (0.855)
Shanghai	GDP (0.982)	Secondary industry (0.980)	Tertiary industry (0.974)	Length of highways (0.954)	Total investment in fixed Assets (0.954)
Jiangsu	Primary industry (0.880)	Secondary industry (0.826)	GDP (0.813)	Length of highways (0.813)	Number of beds in health care agencies (0.802)
Anhui	Total grain products (0.636)	Length of navigable inland waterways (0.598)	Length of railways (0.587)	Primary industry (0.579)	Number of beds in health care agencies (0.480)
Jiangxi	Length of railways (0.918)	Primary industry (0.867)	Length of navigable inland waterways (0.806)	Total grain products (0.804)	Number of higher education institutions (0.794)
Hubei	Length of railways (0.526)	Primary industry (0.511)	Tertiary industry (0.456)	Secondary industry (0.455)	GDP (0.448)
Hunan	Primary industry (0.664)	Tertiary industry (0.580)	GDP (0.579)	Total grain products (0.576)	Secondary industry (0.555)
Zhejiang	Primary industry (0.982)	Number of beds in health care agencies (0.976)	Secondary industry (0.967)	GDP (0.961)	Length of highways (0.955)
Fujian	Primary industry (0.901)	Length of highways (0.856)	Number of beds in health care agencies (0.821)	Tertiary industry (0.819)	GDP (0.815)
Guangdong	Length of highways (0.968)	Primary industry (0.967)	Secondary industry (0.960)	GDP (0.957)	Number of higher education institutions (0.955)
Guangxi	Total grain products (0.745)	Length of navigable inland waterways (0.720)	Length of railways (0.691)	Length of highways (0.652)	Number of beds in health care agencies (0.630)
Sichuan	Total grain products (0.91)	Primary industry (0.72)	Tertiary industry (0.699)	GDP (0.678)	Secondary industry (0.648)
Guizhou	Total grain products (0.922)	Length of railways (0.706)	Length of navigable inland waterways (0..628)	Primary industry (0.544)	Number of higher education institutions (0.429)
Yunnan	Total grain products (0.965)	Length of highways (0.920)	Length of navigable inland waterways (0.898)	Length of railways (0.877)	Primary industry (0.850)
Inner MongoliaInner Mongolia	Total grain products (0.907)	Primary industry (0.800)	Length of navigable inland waterways (0.786)	Length of highways (0.767)	Number of beds in health care agencies (0.767)
Xinjiang	Total grain products (0.930)	Length of highways (0.912)	Length of railways (0.904)	Primary industry (0.888)	GDP (0.879)
Qinghai	Length of navigable inland waterways (0.860)	Primary industry (0.826)	Tertiary industry (0.800)	GDP (0.787)	Length of highways (0.784)
Tibet	Primary industry (0.979)	Total grain products (0.923)	GDP (0.892)	Tertiary industry (0.890)	Number of beds in health care agencies (0.886)
