# Addendum: Spatiotemporal dataset on Chinese population distribution and its driving factors from 1949 to 2013

**DOI:** 10.1038/sdata.2016.78

**Published:** 2016-09-13

**Authors:** Lizhe Wang, Lajiao Chen

**Keywords:** Socioeconomic scenarios, Sustainability, Geography

*Scientific Data* 3:160047 doi: 10.1038/sdata.2016.47 (2016); Published 5 July 2016; Updated 13 September 2016

Since publication, the authors have additionally deposited these data to figshare to further ensure that the data are easily accessible to all researchers:

Wang, L. & Chen, L. *figshare* https://dx.doi.org/10.6084/m9.figshare.c.3291368 (2016).

