# Supplementary material for: Spatiotemporal dataset on Chinese population distribution and its driving factors from 1949 to 2013
Source: Sci Data. 2016 Jul 5;3:160047. doi: 10.1038/sdata.2016.47 (PMC4932880; doi:10.1038/sdata.2016.47)
Supplement: Supplementary Information [file sdata201647-s1.pdf]

Contents of Supplementary Figures

Figure S1………Page 1

Figure S2………Page 2

Figure S3………Page 3

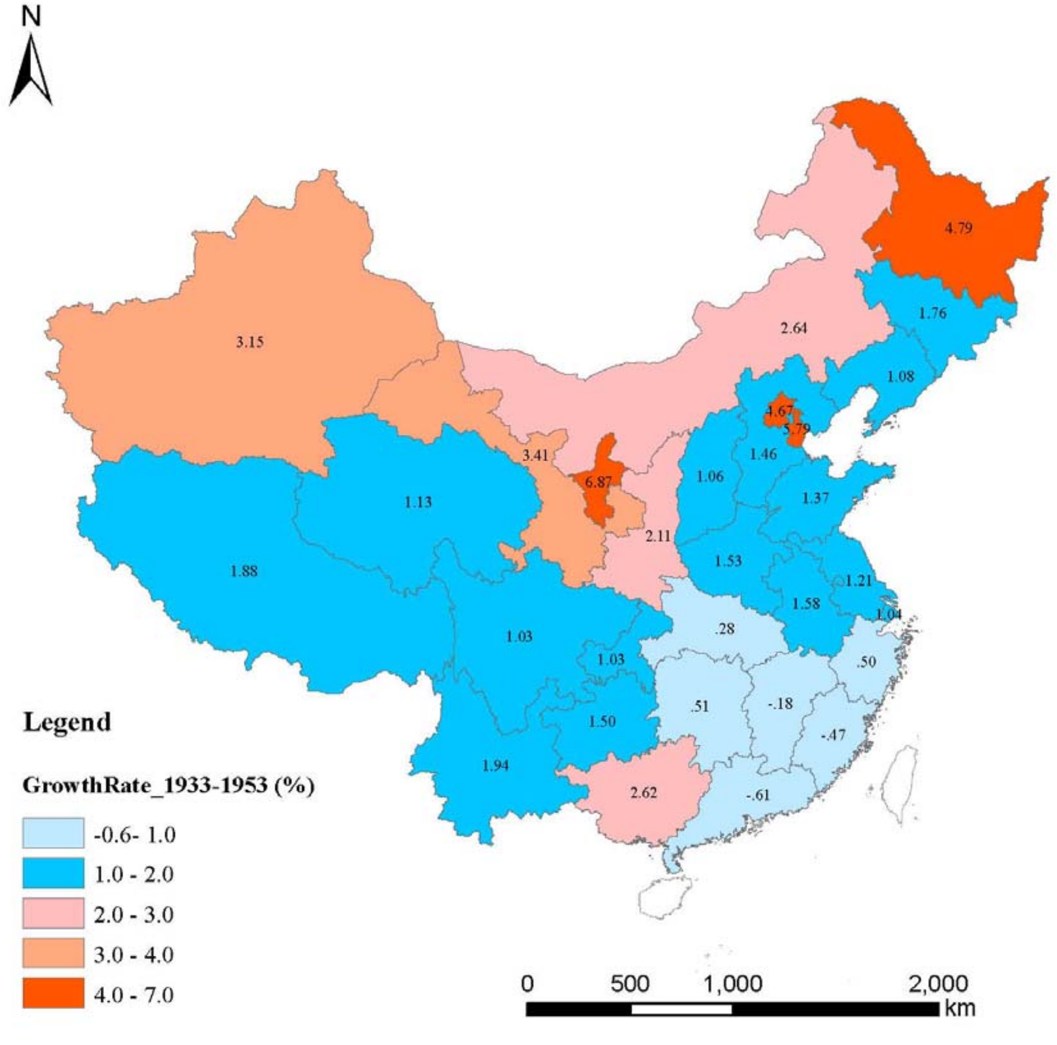

Figure S1 Annual population growth rate (in percentage) of China in 1933-1953 (Taiwan and Hainan are excluded). Different value of population growth rate is expressed by different color group and population of each province is labeled on the map.

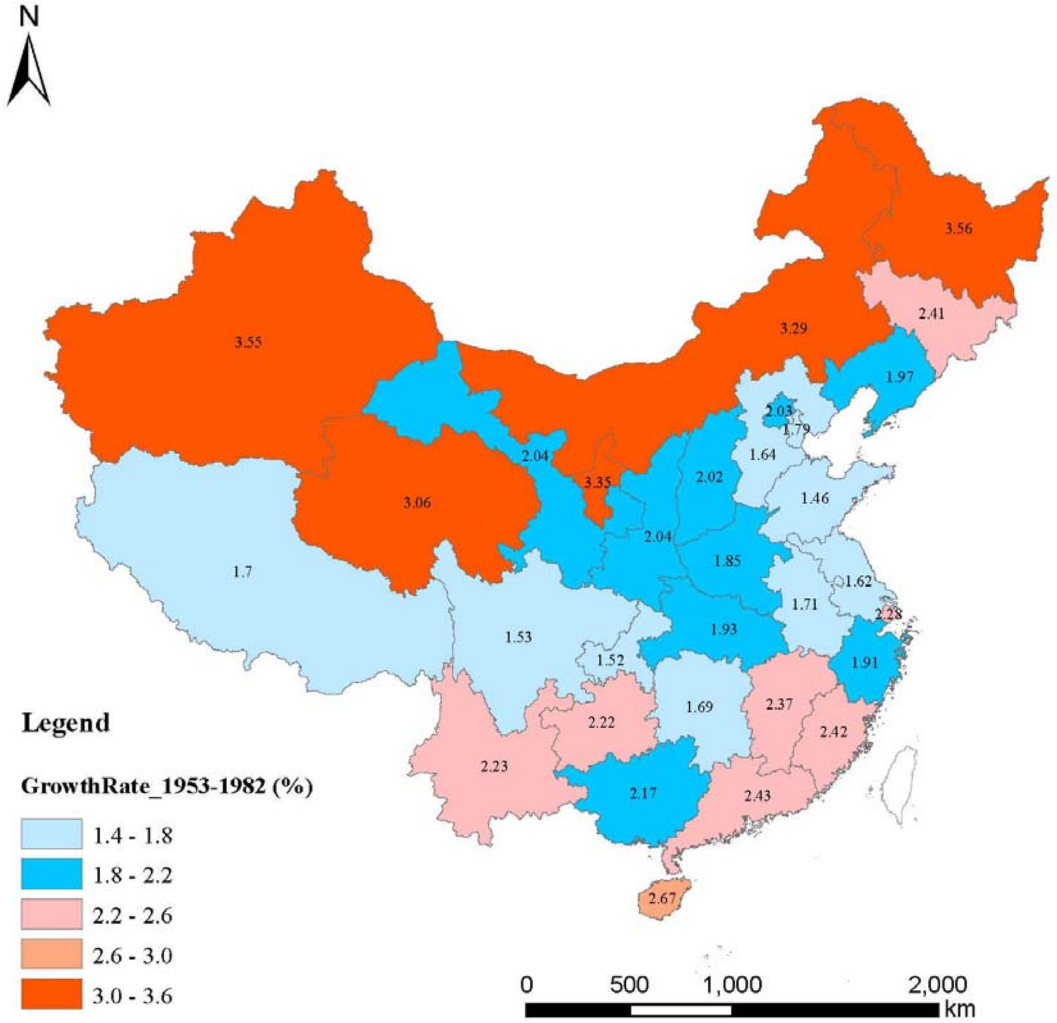

Figure S2 Annual population growth rate (in percentage) of China in 1953-1982 (Taiwan is excluded). Different value of population growth rate is expressed by different color group and population of each province is labeled on the map.

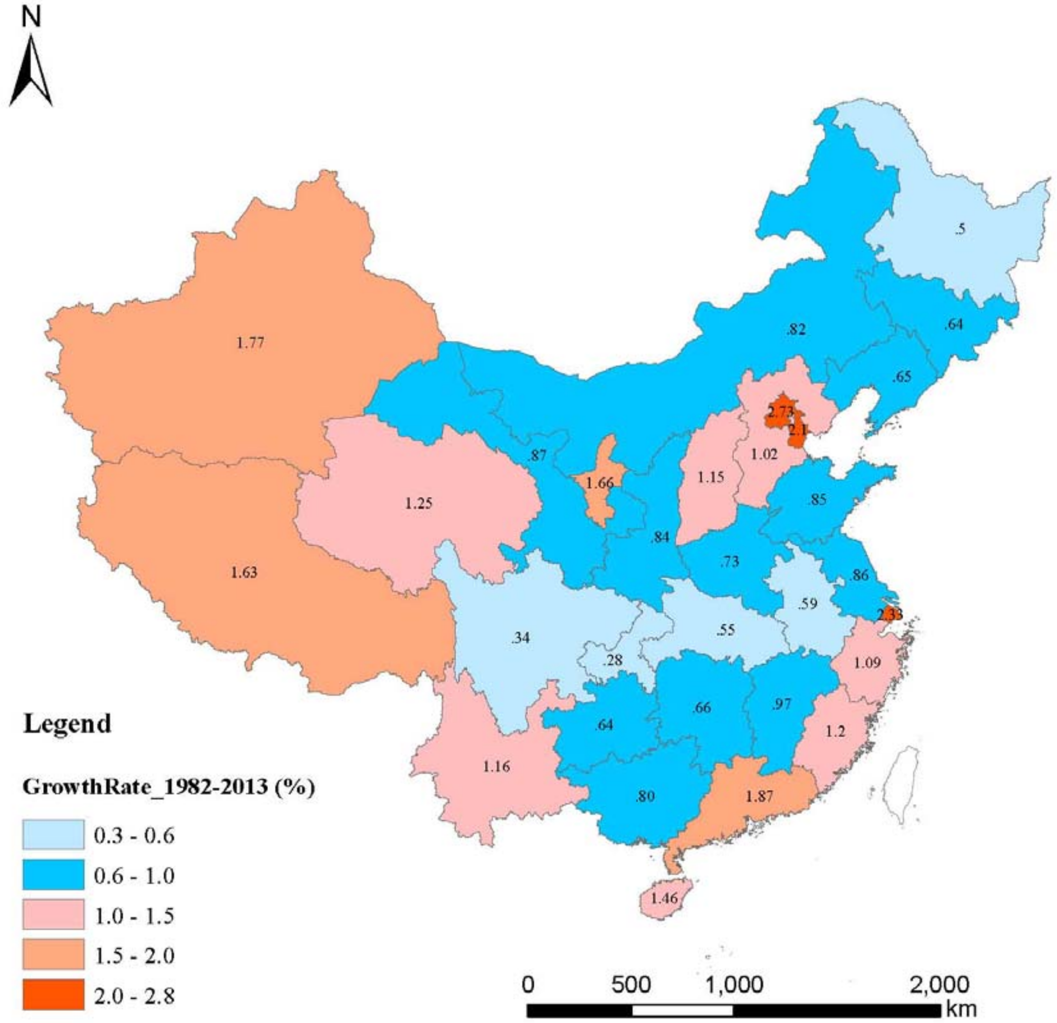

Figure S3 Annual population growth rate (in percentage) of China in 1982-2013 (Taiwan is excluded) Different value of population growth rate is expressed by different color group and population of each province is labeled on the map.
